# Enhanced surgical outcomes in refractory glaucoma: a retrospective study of using adjustable sutures with Ex-PRESS filtration device

**DOI:** 10.3389/fmed.2025.1693402

**Published:** 2025-12-08

**Authors:** Zhen Ji, Dongdong Wei, Na Yu, Xiaolan Yang

**Affiliations:** 1 Department of Ophthalmology, Jinan Second People’s Hospital, Jinan, China; 2 Department of Ophthalmology, Qilu Hospital of Shandong University Dezhou Hospital, Dezhou, China

**Keywords:** refractory glaucoma, Ex-PRESS drainage device, adjustable sutures, intraocular pressure control, filtering bleb morphology

## Abstract

**Purpose:**

Evaluate the clinical efficacy of combining adjustable sutures with the Ex-PRESS Glaucoma Filtration Device in patients with refractory glaucoma, with an emphasis on controlling intraocular pressure (IOP) and reducing postoperative complications.

**Methods:**

This retrospective cohort study involved refractory glaucoma patients treated at Jinan Second People’s Hospital between January 2023 and October 2024. Patients were divided into two groups: one received standard Ex-PRESS implantation (control), and the other received Ex-PRESS implantation with adjustable sutures (experimental). Outcomes included IOP, best-corrected visual acuity (BCVA), filtering bleb morphology, and postoperative complications, assessed over a 6-month follow-up.

**Results:**

The two groups included 75 patients (75 eyes), with 38 (38 eyes) in the experimental group and 37 (37 eyes) in the control group. Both groups showed significant postoperative IOP reduction. The experimental group had higher IOP on Day 1 but significantly lower IOP at all subsequent time points (Week 1 to 6 months, *P* < 0.001). The experimental group had a higher ratio of functioning blebs (84.21% vs. 62.16%, *P* = 0.031) and lower complication rates (5.26% vs. 24.32%, *P* = 0.020). At 6 months after the operation, the two groups did not differ in terms of BCVA (χ^2^ = 2.787, *P* = 0.248). Some complications in the control group (device obstruction and malignant glaucoma) required additional surgical interventions. No cases of endophthalmitis occurred in either group.

**Conclusion:**

The addition of adjustable sutures to Ex-PRESS implantation offers superior IOP control, promotes the formation of functioning bleb, and reduces postoperative complications in refractory glaucoma. This technique provides a promising refinement to existing surgical strategies. Further prospective studies are warranted to confirm long-term benefits.

## 1 Introduction

Glaucoma is a progressive optic neuropathy and the leading cause of irreversible blindness worldwide (1). A particularly challenging variant, refractory glaucoma, fails to respond to standard medical and surgical interventions. This category includes complex cases such as neovascular glaucoma, uveitic glaucoma, and eyes with previously failed filtration surgeries, all of which present formidable therapeutic hurdles. The long-standing gold standard for surgical management has been trabeculectomy, a procedure that creates a new drainage pathway for aqueous humor. However, its long-term success is often compromised by the natural wound-healing process, specifically fibrosis and scarring of the filtration bleb, which leads to surgical failure (2).

To address these limitations, the Ex-PRESS Glaucoma Filtration Device was introduced and received FDA approval in 2002. This miniature, stainless steel shunt is implanted under a scleral flap to divert aqueous humor from the anterior chamber to the subconjunctival space, creating a more standardized and reproducible outflow pathway than a traditional sclerostomy (3). Clinical studies have shown that this minimally invasive approach can achieve higher success rates with comparable safety profiles when compared to trabeculectomy, largely by reducing surgical trauma and inflammation (4). Despite these advantages, the Ex-PRESS device is not without its own challenges. Its highly efficient drainage can lead to early postoperative over-filtration, resulting in complications such as hypotony (dangerously low intraocular pressure, IOP) and a shallow anterior chamber, which can compromise the surgical outcome (5).

A technique that offers a potential solution is the use of adjustable sutures, which have been successfully employed in traditional trabeculectomy to modulate aqueous outflow (6). By securing the scleral flap with releasable or adjustable sutures, surgeons can titrate the flow of aqueous humor non-invasively at the slit lamp in the early postoperative period. This dynamic control of IOP helps mitigate the risks of both over-filtration and under-filtration, thereby improving surgical success rates.

Given the proven utility of adjustable sutures in titrating filtration, it is logical to hypothesize that they could be combined with the Ex-PRESS device to address its primary drawback of early over-filtration. However, the efficacy of this combined approach remains underreported in the literature. Therefore, the present retrospective study aims to investigate the clinical outcomes of using adjustable sutures with the Ex-PRESS Glaucoma Filtration Device in patients with refractory glaucoma. The goal is to provide evidence-based support for a technique that could enhance the safety and success of this important surgical option.

## 2 Materials and methods

### 2.1 Study population

This retrospective cohort study included patients diagnosed with refractory glaucoma (7) who underwent surgical intervention in Jinan Second People’s Hospital between January 2023 and October 2024. The included patients had a confirmed diagnosis of refractory glaucoma (including neovascular glaucoma, secondary glaucoma following intraocular surgery, glaucoma with multiple failed filtration surgeries, traumatic glaucoma, and unknown etiology) and had sufficient anterior chamber depth (≥2.5 mm) for implanting the Ex-PRESS device. Patients were excluded if they (1) had active intraocular inflammation (e.g., uveitis) or endophthalmitis, (2) could not comply with postoperative follow-up, or (3) had concurrent systemic conditions, including severe cardiac, hepatic, or renal dysfunction, pregnancy, lactation, or uncontrolled psychiatric disorders. Eligible patients were divided into two groups based the surgical technique they received. The control group received standard Ex-PRESS implantation, whereas the experimental group received Ex-PRESS implantation with adjustable sutures. The study protocol received ethical approval from the Jinan Second People’s Hospital Ethics Committee (JNEYE20250624) and adhered to the tenets of the Declaration of Helsinki. All participants provided written informed consent prior to surgery.

### 2.2 Surgical procedure

All surgeries were performed by a single, experienced glaucoma surgeon to ensure procedural consistency.

#### 2.2.1 Control group

A fornix-based conjunctival flap was created to expose the sclera. After achieving hemostasis with gentle electrocoagulation, a 4 mm × 3 mm partial-thickness (approximately half to two-thirds) scleral flap was dissected at the surgical limbus. A sponge soaked in mitomycin C (0.4 mg/mL) was placed under the conjunctival and scleral flaps for 2–3 min, followed by copious irrigation with saline solution. A 25-gauge needle was used to create a paracentesis into the anterior chamber, after which the Ex-PRESS Glaucoma Filtration Device (Model P50, Alcon) was implanted under the scleral flap. The flap was secured with two 10-0 nylon sutures, and the conjunctiva was closed with interrupted sutures. Subconjunctival antibiotics and corticosteroids were administered at the conclusion of the surgery.

#### 2.2.2 Experimental group

The procedure was identical to that of the control group except that two adjustable sutures were used for scleral flap closure. Each 10-0 nylon suture was passed from the fornix through the episcleral tissue, entering the sclera 3 mm posterior to the flap bed and exiting through the center of the flap’s edge. The sutures were then tied with a triple-throw slip knot, and the ends were trimmed and left accessible at the fornix. This configuration permitted postoperative adjustment at the slit lamp using microforceps within 1–2 weeks. Suture removal was timed based on standardized criteria incorporating postoperative IOP and anterior chamber depth, as follows: (1) Routine removal (at 1 week): If IOP was between 10 and 21 mmHg and the anterior chamber depth was normal. (2) Early removal (before 1 week): If IOP exceeded 21 mmHg. (3) Postponed removal: In cases of hypotony (IOP < 10 mmHg) or shallow anterior chamber. The exact timing for these cases was determined by the subsequent clinical course.

### 2.3 Outcome measures

Patients were followed for a minimum of 6 months. The primary outcome was IOP, measured using Goldmann applanation tonometry at baseline and at postoperative day 1, week 1, and months 1, 3, and 6. The secondary outcomes, all determined at 6 months, included the following:

1. Best-corrected visual acuity (BCVA), analyzed using logMAR measurements. At the final follow-up, the BCVA of each eye was compared with the baseline level. The changes in final BCVA were categorized as “improved,” “unchanged,” or “decreased.” Clinically significant change was defined as improvement (logMAR decrease more than 0.1), decrease (logMAR increase more than 0.1), or unchange (change less than 0.1 units).
2. Morphology of filtering blebs, evaluated according to the Kronfeld classification system (8) as Type I (microcystic), Type II (diffuse and flat), Type III (absent), or Type IV (encapsulated). Type I and Type II were considered functional.
3. Postoperative complications, which were monitored throughout the follow-up period and treated timely. These included shallow anterior chamber, choroidal detachment, Ex-PRESS device obstruction, malignant glaucoma, and endophthalmitis.

### 2.4 Statistical analysis

Statistical analysis was performed using SPSS 29.0. Continuous variables were initially screened for normality using the Shapiro-Wilk test. Normally distributed variables were expressed as mean ± standard deviation and compared between groups using independent sample *t*-tests. Non-normally distributed variables were expressed as median [P25, P75] and analyzed using the Mann–Whitney *U*-test. Categorical variables (e.g., sex, bleb type, complication rates) were presented as frequencies and percentages (%) and analyzed using the χ^2^ test or Fisher’s exact test, as appropriate. A two-sided *P*-value of <0.05 was considered statistically significant.

## 3 Results

### 3.1 Patients

This study enrolled 75 patients (75 eyes), with 38 (38 eyes) in the experimental group and 37 (37 eyes) in the control group (Table 1). There was no statistically significant difference (*P* > 0.05) between the two groups in age, sex, preoperative IOP, and etiology. Specifically, the experimental group included 29 males and nine females and had an average age of 57.76 ± 16.11 years (range 28–87 years). The mean preoperative IOP was 36.3 (35.4, 41.0) mmHg. The etiologies included neovascular glaucoma (*n* = 15, 39.47%), secondary glaucoma following intraocular surgery (*n* = 11, 28.95%), glaucoma with multiple failed filtration surgeries (*n* = 4, 10.53%), traumatic glaucoma (*n* = 3, 7.89%), and unknown etiology (*n* = 5, 13.16%). The control group included 28 males and nine females and had an average age of 57.49 ± 16.98 years (range 31–84 years). The mean preoperative IOP was 36.7 (34.2, 48.0) mmHg. The etiologies included neovascular glaucoma (*n* = 15, 40.54%), secondary glaucoma following intraocular surgery (*n* = 11, 29.73%), glaucoma with multiple failed filtration surgeries (*n* = 3, 8.11%), traumatic glaucoma (*n* = 2, 5.41%), and unknown etiology (*n* = 6, 16.21%).

**TABLE 1:** Patient characteristics.

Characteristic^†^	Experimental (*n* = 38)	Control (*n* = 37)	*P*-value
Age (year)	57.76 ± 16.11	57.49 ± 16.98	0.935
Sex (*n*, %)	–	–	0.948
Male	29 (76.32%)	28 (75.68%)	–
Female	9 (23.68%)	9 (24.32%)	–
Preoperative IOP (mmHg)	36.3 (35.4, 41.0)	36.7 (34.2, 48.0)	0.949
Operated eye	–	–	0.404
Left (OS)	18 (47.37%)	14 (37.84%)	–
Right (OD)	20 (52.63%)	23 (62.16%)	–
Etiology	–	–	0.981
Neovascular glaucoma	15 (39.47%)	15 (40.54%)	–
Secondary glaucoma following intraocular surgery	11 (28.95%)	11 (29.73%)	–
Multiple failed filtration surgeries	4 (10.53%)	3 (8.11%)	–
Traumatic glaucoma	3 (7.89%)	2 (5.41%)	–
Unknown	5 (13.16%)	6 (16.21%)	–

† Expressed as mean ± standard deviation, median [P25, P75], or count (ratio).

### 3.2 Outcomes

The experimental group and the control group had indistinguishable preoperative IOP (36.3 vs. 36.7 mmHg, *P* = 0.949), and for both groups the IOP dropped after the surgery (Table 2). The experimental group had a significantly higher IOP than the control group on postoperative day 1 (15.7 vs. 12.0 mmHg, *P* < 0.001) but significantly lower IOP at all later time points: Week 1, 13.2 vs. 15.0 mmHg, *P* < 0.001; 1 month, 15.0 vs. 17.0 mmHg, *P* < 0.001; 3 months, 16.5 vs. 20.0 mmHg, *P* < 0.001; 6 months, 18.3 vs. 21.5 mmHg, *P* < 0.001. In addition, the IOP remained at a low level throughout the follow-up period in the experimental group, whereas in the control group, it increased from 12.0 mmHg on Day 1 to 21.5 mmHg at 6 months. As to IOP reduction from baseline, the experimental group achieved a significantly greater percentage reduction compared to the control group at the 6-month endpoint (51.8% vs. 41.9%, *P* = 0.002). This indicates that the experimental procedure was more effective in lowering IOP throughout the 6-month follow-up period. A *post hoc* power analysis was conducted for the week 1 IOP comparison using the Mann-Whitney *U*-test. With sample sizes of 38 and 37 in the experimental and control groups, a standard deviation of 1.93, the analysis demonstrated a statistical power of 98.6% (α = 0.05) to detect a significant difference between the group medians (13.2 vs. 15.0). This indicates a high probability that the study was capable of detecting the observed effect.

**TABLE 2:** Comparison of intraocular pressure at different time points.

Time	Experimental (*n* = 38)^†^	Control (*n* = 37)^†^	Difference (95%CI)^&^	*P*-value
Pre-surgery	36.3 (35.4, 41.0)	36.7 (34.2, 48.0)	0.0 (−2.2, 3.0)	0.949
Day 1	15.7 (14.0, 17.7)	12.0 (8.7, 13.3)	−4.4 (−5.7, −3.0)	<0.001
Week 1	13.2 (12.3, 14.6)	15.0 (14.3, 16.0)	1.8 (1.0, 2.6)	<0.001
1 month	15.0 (14.2, 16.1)	17.0 (16.3, 18.2)	2.0 (1.7, 2.7)	<0.001
3 months	16.5 (16.0, 17.3)	20.0 (18.7, 20.7)	3.0 (2.5, 3.7)	<0.001
6 months	18.3 (17.2, 19.3)	21.5 (21.0, 23.0)	3.7 (2.8, 4.3)	<0.001
IOP reduction*	51.8 ± 9.4	41.9 ± 15.4	9.8 ± 3.0	0.002

† Expressed as median [P25, P75]. Unit: mmHg. *Expressed as mean ± standard deviation. Unit: %. ^&^Difference calculated using Hodges-Lehmann estimation.

At 6 months after the operation, the two groups did not differ in terms of BCVA (χ^2^ = 2.787, *P* = 0.248). However, the experimental group had a higher incidence of improvement (26.32% vs. 16.22%) and a lower incidence of deterioration (2.63% vs. 10.81%), as shown in Table 3. The experimental group had a significant higher ratio of functioning filtering blebs compared to the control group (84.21% vs. 62.16%, *P* = 0.031, Table 4), and it also had a significantly lower incidence of complications (5.26% vs. 24.32%, χ^2^ = 5.442, *P* = 0.020, Table 5). The complications in the experimental group included transient shallow anterior chamber (2, 5.26%) and choroidal detachment (1, 2.63%), and all were resolved after conservative treatment. The complications in the control group included shallow anterior chamber (7, 18.92%), choroidal detachment (2, 5.41%), drainage tube obstruction (1, 2.70%), and malignant glaucoma (1, 2.70%). Conservative options succeeded in resolving only the first two complications. The drainage tube blockage was finally resolved by Nd:YAG laser opening. The malignant glaucoma was resolved by anterior vitrectomy with scleral flap reinforcement and anterior chamber reformation. There was no endophthalmitis in either group.

**TABLE 3:** Change in best-corrected visual acuity.

Visual change	Experimental (*n* = 38)^†^	Control (*n* = 37)^†^
Improved	10 (26.32%)	6 (16.22%)
Unchanged	27 (71.05%)	27 (72.97%)
Decreased	1 (2.63%)	4 (10.81%)

† Expressed as case count (percentage). χ^2^ = 2.787, *P* = 0.248.

**TABLE 4:** Morphology of filtering bleb.

Type	Experimental (*n* = 38)^†^	Control (*n* = 37)^†^
I	11 (28.95%)	8 (21.62%)
II	21 (55.26%)	15 (40.54%)
Functional (I + II)^§^	32 (84.21%)	23 (62.16%)
III	3 (7.90%)	5 (13.51%)
IV	3 (7.90%)	9 (24.32%)

† Expressed as case count (percentage). ^§^ χ^2^ = 4.660, *P* = 0.031.

**TABLE 5:** Complications.

Complication	Experimental (*n* = 38)^†^	Control (*n* = 37)^†^
Shallow anterior chamber	2 (5.26%)	7 (18.92%)
Choroidal detachment	1 (2.63%)	2 (5.40%)
Drainage tube obstruction	0 (0.00%)	1 (2.70%)
Malignant glaucoma	0 (0.00%)	1 (2.70%)
Total^§^	2 (5.26%)	9 (24.32%)

† Expressed as case count (percentage). ^§^ χ^2^ = 5.442, *P* = 0.020.

## 4 Discussion

Refractory glaucoma presents significant therapeutic challenges due to underlying pathophysiology, such as neovascularization or scarring from previous surgeries, which compromises the success of traditional filtration procedures. The Ex-PRESS Glaucoma Filtration Device offers a minimally invasive alternative by creating a standardized, reproducible outflow channel to divert aqueous humor from the anterior chamber to the subconjunctival space. The device features an external flange to prevent intrusion into the anterior chamber and a spur to prevent extrusion; this design is intended to enhance surgical safety compared to traditional trabeculectomy (9). While its efficacy has been confirmed by numerous studies (10–13), the risk of early postoperative over-filtration—leading to complications like hypotony and shallow anterior chamber—remains a significant clinical concern (14).

The use of adjustable sutures with Ex-PRESS implantation directly addresses this key limitation. By securing the scleral flap with adjustable sutures, surgeons can achieve higher initial outflow resistance, which can then be titrated postoperatively. If the initial IOP is too high, sutures can be loosened or removed at the slit lamp to increase aqueous drainage. This dynamic modulation allows for a more controlled and stable postoperative course, mitigating the risks associated with both over- and under-filtration (15).

Our findings strongly support this mechanism. The experimental group had a significantly higher IOP on postoperative Day 1, which is consistent with tighter initial scleral flap closure. This controlled resistance likely prevented the early hypotony and shallow anterior chambers that were more prevalent in the control group. The subsequent IOP decreased slightly one week after surgery corresponds temporally with the removal of the adjustable suture and the overflow of aqueous humor from the scleral flap. IOP in the experimental group stabilized at a lower level than in the control group at all follow-up points (1 week to 6 months). This superior long-term control is attributable to the ability to titrate filtration, which leads to the formation of a more durable, functional filtering bleb. These results align with the findings of Qu et al. (16), who also concluded that the combined technique is safe and effective in treating refractory glaucoma.

Regarding visual outcomes, although there was no statistically significant difference in final BCVA between the groups, the experimental group showed a favorable trend, with a higher proportion of patients experiencing vision improvement and a lower proportion with vision deterioration. This observation may be linked to the quality of IOP control. The stable, lower long-term pressures achieved in the experimental group are crucial for protecting the optic nerve from further glaucomatous damage. In contrast, the progressive rise in IOP seen in the control group after the first month suggests ongoing stress on the optic nerve, which potentially limits the extent of visual recovery.

Functioning filtering blebs are the anatomical cornerstone of successful filtration surgery (17). As this is a retrospective study, a significant portion of the data could not be reassessed with more modern methods. Therefore, adopting the Kronfeld classification was a necessary decision to maintain methodological consistency across the entire dataset. This study found a significantly higher rate of functioning blebs (Kronfeld Types I and II) in the experimental group. Previous studies also reported that the proportion of functional filtering blebs was significantly higher following trabeculectomy with adjustable sutures compared to the procedure alone (18). Blebs in adjustable suture group were more diffuse, low lying and presented a more ideal vascularity (19). The likely mechanism is that by preventing early postoperative hypotony, the adjustable sutures reduce the associated inflammation and fibrin leakage, which are known precursors to bleb fibrosis and failure. The diffuse, microcystic blebs seen more frequently in the experimental group facilitate sustained aqueous drainage and stable IOP control. Conversely, the higher rate of scarred or encapsulated blebs in the control group correlated with the observed treatment failure and rising IOP over time. For refractory type IV filtering blebs, a subconjunctival injection of 5-fluorouracil (5-FU; 5 mg) was administered daily. If bleb function failed to improve after three consecutive injections, bleb needling was performed. Post-intervention, bleb morphology was monitored closely, and IOP-lowering medications were augmented as necessary. Patients were advised of the potential need for future surgical intervention.

Currently, the main types of Ex-PRESS drainage devices used in clinical practice are the P50 and P200. Studies have reported that both types exhibit comparable efficacy in lowering IOP; however, the P50 is associated with a lower incidence of postoperative complications such as hypotony and shallow anterior chamber compared to the P200 (20). This may be attributed to the larger internal diameter of the P200, which results in a higher flow rate and lower resistance to aqueous outflow. In our study, the P50 type was used. The experimental group had a significantly lower overall complication rate. By providing precise, early control of aqueous outflow, adjustable sutures directly reduce the incidence of hypotony-related complications like shallow anterior chamber and choroidal detachment. The cases of shallow anterior chamber (2, 5.26%) and choroidal detachment (1, 2.63%) in the experimental group were transient and resolved with conservative management. In contrast, the control group experienced a higher complication burden, with some cases requiring additional surgical intervention. Kanner et al. (21) found that of 345 cases of Ex-PRESS implantation, device obstruction was the most common complication (6 eyes, 1.74%), yet the control group had a higher incidence of device obstruction (1, 2.70%). The obstruction was not cleared by conservative treatment and was treated successfully with an Nd:YAG laser to disrupt a fibrous membrane, thus restoring bleb function. Another patient in the control group developed malignant glaucoma, a severe complication of intraocular surgery characterized by aqueous misdirection. This condition is often triggered by postoperative hypotony and a shallow or collapsed anterior chamber, which can initiate a cascade of events leading to a pressure-dependent posterior-to-anterior fluid shift (22). Although multiple patient-specific factors may have contributed, the initial hyperfiltration in the control group was a likely inciting event. This case ultimately required anterior vitrectomy and scleral flap revision, which underscores the severity of complications that can arise from uncontrolled filtration.

No cases of endophthalmitis occurred in either group, consistent with the low infection rates reported for Ex-PRESS procedures (5, 23, 24). However, the procedure is not without other long-term risks. Surgeons must ensure proper placement, as contact between the device and the corneal endothelium can lead to rapid cell loss and corneal decompensation (25, 26). Recent studies have also suggested a potential association with epiretinal membrane formation (27), but this was not observed in our study, likely due to the limited follow-up period.

## 5 Conclusion

In conclusion, within the constraints of a 6-month follow-up, our study suggests that combining the Ex-PRESS device with adjustable sutures offers short-term advantages over standalone surgery in patients with refractory glaucoma. These include superior IOP control, more functional filtering blebs, and a lower postoperative complication rate. The adjustable suture technique, therefore, appears to be a valuable refinement for enhancing early surgical outcomes in this challenging population.

However, the abbreviated follow-up period inherently limits our ability to assess critical long-term endpoints such as bleb survival, IOP stability, and late-onset complications. Consequently, the long-term efficacy and safety of this approach remain to be determined. Future prospective, multi-center studies with larger cohorts and extended follow-up are essential to validate and build upon these preliminary findings.

## Data Availability

The original contributions presented in this study are included in this article/supplementary material, further inquiries can be directed to the corresponding author.
